# Comparing Austin Moore and Bipolar Prostheses for Management of Femoral Neck Fractures in Low-Resource Hospitals in Rural India

**DOI:** 10.7759/cureus.75205

**Published:** 2024-12-06

**Authors:** Kapil Shinde, Rohit S Kumar, Prashant Awasthi, Tarik Al-Dahan, Emadeldin Ahmed, Sravan Sanka, Muhammad Asad Arif, Salih Seidahmed

**Affiliations:** 1 Trauma and Orthopaedics, Queen Elizabeth The Queen Mother Hospital, Margate, GBR; 2 Trauma and Orthopaedics, William Harvey Hospital, Ashford, GBR

**Keywords:** austin moore prosthesis, bipolar prosthesis, efficient healthcare, fracture neck femur, health economic analysis, low resource setting, prosthesis comparison

## Abstract

Background: Femoral neck fractures in elderly individuals cause significant morbidity, and their management is particularly challenging in rural areas where healthcare access is limited. The recommended treatment for displaced femoral neck fractures in elderly patients with poor mobility, cognitive dysfunction and multiple comorbidities is a hemiarthroplasty, which can be performed with various implants, including monopolar implants like Austin Moore prosthesis (AMP) and bipolar prosthesis (BP). In developing countries like India, rural areas often have constraints with healthcare resources. Furthermore, the per-capita income is low, limiting access to affordable healthcare. As a result, treatment is often tailored to ensure affordability, and AMP continues to be used as it is a relatively inexpensive implant. The objective of our study is to assess and compare the mortality, infection rate and functional outcomes (Harris hip score [HHS]) of AMP and BP in treating femoral neck fractures one year following surgery in a resource-constrained setting in a rural district general hospital in India.

Methodology: This retrospective observational study analysed all patients who underwent a hemiarthroplasty for acutely displaced femoral neck fractures between 1 January 2017 and 31 December 2017, with a minimum one-year follow-up following surgery. Pathological hip fractures, patients with pre-existing hip pathologies and those with an abbreviated mental test score of six or less were excluded. Medical records were reviewed, and demographic data, mortality, infection rates and HHS one year following surgery were recorded and compared for patients who underwent hemiarthroplasty with an AMP and BP.

Results: A total of 118 patients underwent hemiarthroplasty, with two (1.69%) lost to follow-up. Therefore, 116 patients were included, comprising 81 (69.83%) women and 35 (30.17%) men, with similar demographics between both groups. No statistically significant difference was found in mortality rate (AMP 1, 1.79%, vs. BP 1, 1.67%, *P *= 0.96), infection rate (AMP 1, 1.82%, vs. BP 1, 1.69%, *P *= 0.96) and HHS (AMP 85.2 vs. BP 88.5; *P *= 0.08). No dislocations or periprosthetic fractures were noted at one-year follow-up in both groups.

Conclusions: While AMP and BP have similar clinical and functional outcomes, AMP is more cost-effective and perhaps more suitable in low socioeconomic demographics and low-resource settings. Further research is suggested to evaluate long-term outcomes in underserved populations with a low per-capita income.

## Introduction

It is estimated that the annual incidence of hip fractures in India is about 600,000, and this number is expected to increase significantly due to an ageing population and increased life expectancy [1]. Hemiarthroplasty is recommended as a treatment for elderly patients with cognitive dysfunction, multiple comorbidities or sedentary lifestyles [2]. Hemiarthroplasty can be performed using a monopolar prosthesis such as an Austin Moore prosthesis (AMP) and a bipolar prosthesis (BP) [2]. BP are noted to be two to five times more expensive than monopolar implants [2].

India’s healthcare system predominantly operates on a cash-based model, where most expenses are out-of-pocket and directly borne by patients. The country’s per-capita income, approximately $2,400 as of 2023, severely limits the ability of lower-income populations to access advanced medical care [3]. Health insurance coverage is low, and there is a lack of access to quality healthcare services, especially in rural areas [4-5]. Rural hospitals are low-resource settings with limited infrastructure and equipment [4-5]. Hip fractures place a significant financial burden on individuals seeking orthopaedic procedures, and financial constraints result in them often choosing less expensive treatment options despite potentially better alternatives.

In countries with limited healthcare resources and a low per-capita income, clinicians often need to consider interventions with acceptable clinical outcomes that are cost-effective. The primary aim of this study is to compare the functional outcomes, one year following treatment for femoral neck fractures with hemiarthroplasty using AMP versus BP. The secondary aim is to compare infection and mortality rates a year following hemiarthroplasty with AMP versus BP.

## Materials and methods

This retrospective observational study was conducted in the orthopaedics department of a district civil hospital in rural central west India, serving a population predominantly engaged in agricultural work, with limited access to advanced medical facilities. The study was approved by the institutional ethics committee (IRB number IRB/2016/RCHMMC-114).

All patients 65 years and older who underwent primary hemiarthroplasty for acutely displaced femoral neck fractures between 1 January 2017 to 31 December 2017, with a minimum of one-year post-operative follow-up, were included in the study. We used the Garden classification [6] to determine if a fracture was displaced. Patients with pathological hip fractures and pre-existing hip pathologies were excluded. We also excluded patients with cognitive dysfunction identified by an Abbreviated Mental Test Score [7] less than six as the collection of functional outcomes in this cohort would have been inaccurate. 

All patients were operated by experienced surgeons, using the same surgical approach, i.e., anterolateral approach to the hip and had the same pre-operative and post-operative care as per local protocols. Both AMP and BP were used and the decision to choose one over the other was based on surgeon discretion. The AMP is a monoblock prosthesis and was implanted uncemented using the technique described by Austin Moore [8]. All BP used in our series were cemented Self-Centering™ Bipolar prostheses manufactured by DePuy Synthes (Warsaw, IN), and implantation was performed as described in the surgical technique manual [9]. 

Patient medical records were reviewed, and baseline demographics such as patient age at the time of surgery and sex were collected. The Harris Hip score (HHS), which is a validated tool for the assessment of post-operative outcomes after arthroplasty of the hip [10], was recorded for both groups one year following surgery, and the standard deviation (SD) was calculated to establish 95% confidence intervals. SPSS version 24 for Windows (IBM Corp., Armonk, NY) was used for analyzing data. An independent t-test was used to calculate the *P*-value to assess if there was statistical significance in HHS between both groups. Medical records were reviewed, and mortality and infection rates at one year for both groups were studied. Mortality and infection rates between both groups were compared using Pearson's chi-square test. All statistical tests in this study were considered significant if a *P*-value <0.05 was recorded.

## Results

A total of 118 patients with displaced femoral neck fracture were included in the study of which 2 (1.69%) were lost to follow-up at one year. Hence, 116 patients were included in the study. The study population included 81 (69.83%) women and 35 (30.17%) men, with the age ranging from 65 to 85 years. Most patients were elderly women, reflecting the higher incidence of osteoporotic fractures in this demographic. The demographic characteristics of the AMP and BP groups, including age, sex and comorbidities were similar.

Two (1.72%) patients did not survive at the end of one year. Table 1 shows there was no statistical significance (*P *< 0.05) in comparing the mortality rate between both groups. 

**Table 1:** Mortality rate at one year following hemiarthroplasty with AMP and BP. The *P*-value was calculated using the chi-square test, with *P* < 0.05 considered significant. AMP, Austin Moore prosthesis; BP, bipolar prosthesis

Variable	Total	AMP	BP	P-value	Chi-square value
Patients (*n*)	116	56 (48.28%)	60 (51.72%)		
Deceased	2 (1.72%)	1 (1.79%)	1 (1.67%)	0.96	0.002

As one patient in each group did not survive for one year, the sample sizes for calculating the infection rate and HHS at one year were 55 for the AMP group and 59 for the BP group. The study reported similar post-operative infection rates between the AMP and BP groups. The infection rate was 1 (1.82%) for the AMP group compared to 1 (1.69%) for the BP group. Table 2 shows that the difference in infection rates was not statistically significant. All infections were superficial infections treated with antibiotics.

**Table 2:** Infection rate at one year following hemiarthroplasty with AMP and BP. The *P*-value was calculated using the chi-square test, with *P* < 0.05 considered significant. AMP, Austin Moore prosthesis; BP, bipolar prosthesis

Variable	Total	AMP	BP	P-value	Chi-square value
Patients (*n*)	114	55 (48.25%)	59 (51.75%)		
Infection rate	2 (1.75%)	1 (1.82%)	1 (1.69%)	0.96	0.002

After one year, the BP group had a slightly higher mean HHS of 88.5, compared to 85.2 for the AMP group. Table 3 shows that this difference was not statistically significant (*P* = 0.08). 

**Table 3:** HHS in patients at 12 months following hemiarthroplasty with AMP or BP. The *P*-value was calculated using an independent *t*-test, with *P* < 0.05 considered significant. AMP, Austin Moore prosthesis; BP, bipolar prosthesis; HHS, Harris Hip score

Implant	Sample size	Mean	Standard deviation	*P*-value	*t*-score
AMP	55 (48.25%)	85.2	5.7	0.08	-3.23
BP	59 (51.75%)	88.5	5.2		

No prosthetic hip joint dislocation or periprosthetic fractures were seen at the one-year follow-up in our cohort. 

## Discussion

**Figure 1:**
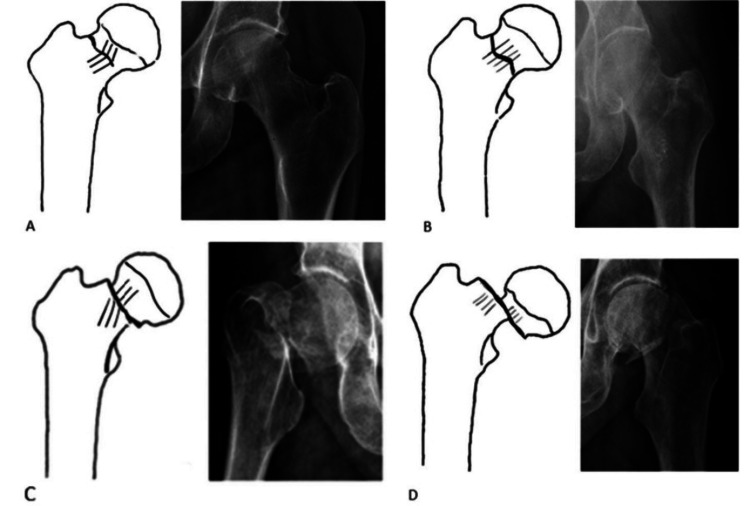
Garden's classification for femoral neck fractures. Reproduced from Kazley et al. [6], with permission from Clinical Orthopaedics and Related Research.

Treatment options for displaced femoral neck fractures, i.e., Garden [6] types III and IV fractures in the elderly, include hemiarthroplasty (either AMP or BP) or total hip arthroplasty [2]. Figure 1 shows the Garden [6] classification of femoral neck fractures. Hemiarthroplasty is recommended for elderly, frail patients with multiple comorbidities who lead a sedentary lifestyle [2]. 

In our study, the functional score (HHS), infection rate and mortality rate of AMP and BP at the end of 12 months were similar, and there was no statistical significance in comparison between the two groups. A review of the literature too suggests that bipolar prosthesis has similar outcomes compared with unipolar prostheses while comparing blood loss, blood transfusion, hospital stay, mortality, complication, reoperation, dislocation and HHS [11,12]. Bipolar prosthesis may offer a better range of motion and a lower rate of acetabular erosion [11,12]. Mishra et al. [13] compared AMP and BP in Nepal, which is a low per-capita income country like India. Their study showed that AMP and BP had no statistical differences in complication rate, acetabular erosion and HHS [13]. While AMP had a higher HHS in their study, it was not statistically significant [13]. 

In our study, we identified that AMP ($35) was nearly 18 times cheaper than a BP ($660). Other studies too confirm that BP is more expensive than monopolar implants [2]. Given that clinical outcomes were similar between both groups in our study and other studies [11-13], AMP may be a better option in resource-limited settings, where cost-effectiveness is a key consideration. However, BP may offer added benefits in specific cases like patients with high activity levels, better bone quality and younger patients in whom the increased range of motion and lower risk of acetabular wear will be beneficial [11,12].

In the United Kingdom, the National Institute for Health and Care Excellence (NICE) recommends the use of a proven stem instead of an AMP [14]. However, in countries with low per- capita income like India, it may be difficult to offer evidence-based treatment due to constraints on the availability of resources. Furthermore, evidence-based treatment may not be financially viable for a significant portion of the population, and hence, treatment must be tailored to ensure affordability with optimal outcomes. Treating femoral neck fractures in a rural, low-resource setting is challenging, but we have demonstrated that acceptable outcomes can be achieved to facilitate a sustainable health economic model, and this model can be replicated in other low-income and low-resource countries.

This study has several limitations. First, this is a retrospective study with a relatively small sample size and the single-centre design may limit the generalizability of the findings. Furthermore, patient outcomes were only reviewed up to one year following surgery, and hence, long-term outcomes and complications such as aseptic loosening could not be studied. These limitations should be considered when interpreting the results. While anecdotally patients in both groups expressed similar satisfaction with pain relief and mobility, including a validated satisfaction survey in future studies could provide insights into whether BP’s minor functional advantage translates to greater patient satisfaction, potentially supporting its use in specific cases. Finally, both prostheses were offered to patients at the surgeon's discretion without any randomization. It is possible that BP was preferentially offered to younger patients who had good mobility before the fracture and were physically fit, while AMP was offered to elderly, frail patients with pre-existing comorbidities and poor mobility before the injury. We did not review the American Society of Anaesthesiologists (ASA) physical classification system which is a surrogate for patient fitness before surgery [15]. It would be useful to review the ASA score in future studies to ensure that both cohorts have similar characteristics.

Future research with large sample size, long follow-up period, patient satisfaction outcomes and a review of additional parameters such as the ASA score is necessary to fully assess the long-term outcomes and cost-effectiveness of both prosthetic options in this patient population.

## Conclusions

Our study demonstrates that good functional outcomes can be achieved with both AMP and BP for the treatment of femoral neck fractures. While BP offers slightly better functional outcomes in the management of femoral neck fractures, the differences are not statistically significant and its higher cost may be a limiting factor in its utilization in low-income countries. While we are unable to recommend one prosthesis over the other due to similar clinical and functional outcomes, the AMP can be considered in low socio-economic demographics due to its advantages in achieving a sustainable health economic model. While AMP is being phased out in developed countries, it continues to be a versatile implant and a pragmatic choice for the management of femoral neck fractures in the elderly in low-income countries with limited access to resources.

The long-term outcomes and complications of both prostheses warrant further investigation. Additional research with larger sample sizes and extended follow-up periods is necessary to fully evaluate the long-term efficacy and overall cost-effectiveness of both the AMP and BP in the management of femoral neck fractures in similar low-resource settings.
